# A case report of anal fistula-associated mucinous adenocarcinoma developing 3 years after treatment of perianal abscess

**DOI:** 10.1186/s40792-023-01743-3

**Published:** 2023-09-11

**Authors:** Michihiro Koizumi, Akihisa Matsuda, Takeshi Yamada, Koji Morimoto, Itaru Kubota, Yawara Kubota, Shuzo Tamura, Kenta Tominaga, Takashi Sakatani, Hiroshi Yoshida

**Affiliations:** 1 Nishiarai Coloproctology Clinic, 3-7-13 Shimane, Adachi-ku, Tokyo, Japan; 2 Department of Gastrointestinal and Hepato‐Biliary‐Pancreatic Surgery, Nippon Medical School, Sendagi 1-1-5, Bunkyo-ku, Tokyo, 113-8603 Japan; 3 Department of Diagnostic Pathology, Nippon Medical School, Sendagi 1-1-5, Bunkyo-ku, Tokyo, 113-8603 Japan

**Keywords:** Fistula-associated mucinous adenocarcinoma, Anal carcinoma, Anal fistula, Perianal abscess, Cryptitis, Cryptoglandular infectious theory, Transanal ultrasonography, Carcinogenesis, Dysplasia

## Abstract

**Background:**

A long-standing (over 10 years) anal fistula is considered a fundamental cause of fistula-associated mucinous adenocarcinoma (FAMC). Perianal abscesses and anal fistulas are two sequential phases of the same anorectal infectious process. We experienced a case of FAMC which developed 3 years after the treatment of a perianal abscess.

**Case presentation:**

A 68-year-old woman was admitted to our hospital because of progressive anal pain and a palpable tumor. She had a history of undergoing a drainage operation for a perianal abscess 3 years previously. A 15 × 15-mm tumor at the former drainage site was identified; transanal ultrasonography showed an intersphincteric fistula connecting to the tumor. A biopsy taken from the tumor demonstrated mucinous adenocarcinoma; the tumor was diagnosed as FAMC. Laparoscopic abdominoperineal resection was performed. Histopathology showed highly dysplastic cells lining the lumen of the anal fistula and poorly differentiated mucinous adenocarcinoma proliferating in the dermis and epidermis in the distal aspect of the fistula.

**Conclusions:**

FAMC can develop within fewer than 3 years after the development of a perianal abscess and anal fistula.

## Background

Chronic inflammation in organs is one of the causes of cancer development and proliferation [1, 2]. A long-standing (over 10 years) anal fistula is considered an etiology of fistula-associated mucinous adenocarcinoma (FAMC) [3–5].

However, we encountered a patient with FAMC arising 3 years after the treatment of a perianal abscess. Perianal abscesses and anal fistulas are two sequential phases of the same anorectal infectious process. In this case, the process of carcinogenesis and proliferation in this short period was inexplicable.

## Case presentation

**Fig. 1:**
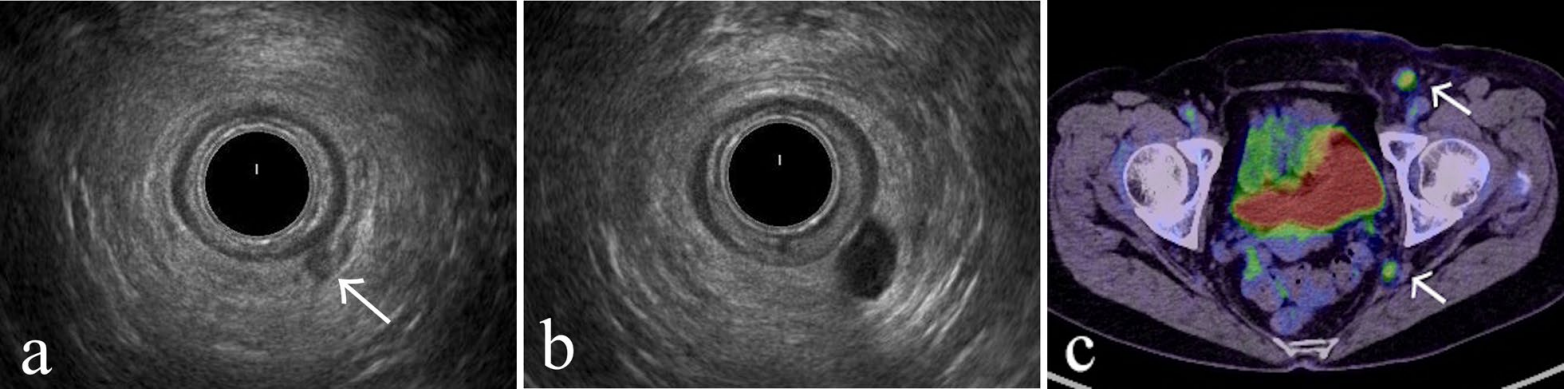
Imaging studies. **a** Transanal ultrasonography revealed a fistula-like structure with unclear boundaries in the intersphincteric layer at the time of the perianal abscess 3 years previously (arrow). **b** Anal fistula was expanded with hypoechoic content at the time FAMC was diagnosed. **c** [18F]-Fluoro-2-deoxy-d-glucose (FDG)–positron emission tomography/computed tomography showed lymphadenopathy in the right internal iliac and inguinal regions with abnormal FDG accumulation (arrows)

A 68-year-old woman presented to our institution because of increasing anal pain and a palpable tumor. She did not have other gastrointestinal symptoms, including inflammatory bowel disease. She had a history of a perianal abscess at the 5 o’clock position 3 years previously. Transanal ultrasonography showed a fistula-like structure (Fig. 1a) connecting to the abscess. The perianal abscess was treated with a drainage procedure under local anesthesia in the outpatient setting. Normal pus was confirmed without any tumor findings. Her symptoms were resolved 3 days later, but she did not come to the hospital for a follow-up visit thereafter.

**Fig. 2:**
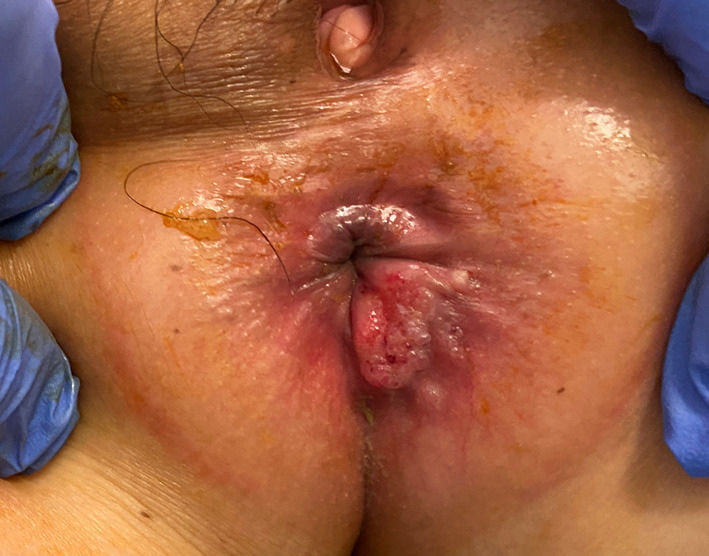
Preoperative photograph. The tumor was observed at the 5 o’clock position, corresponding to the drainage site for the perianal abscess

**Fig. 3:**
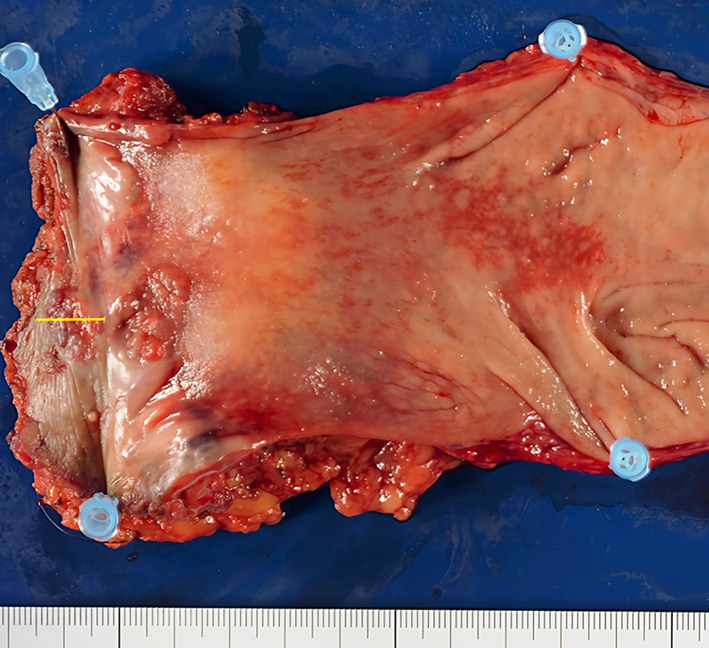
Image of the resected specimen. The secondary opening of the fistula was obscured, because it was involved in the tumor. The yellow bar indicates a cross section of the pathological image

**Fig. 4:**
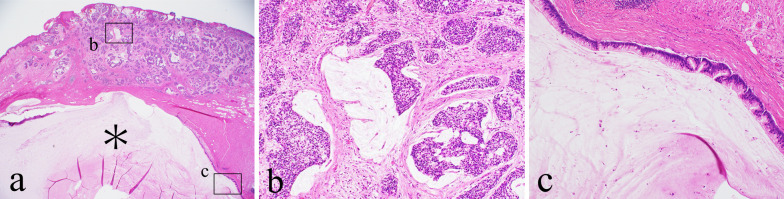
Histopathologic photographs, hematoxylin and eosin (H&E) staining. **a** Lumen of the fistula (*) is expanded with a massive mucinous component. Carcinoma cells invade the dermis and epidermis around the distal site of the fistula (×40). **b** Mucin-containing poorly differentiated adenocarcinoma is present (×100). **c** Highly dysplastic epithelial cells lined the lumen of the anal fistula (×100)

Transanal ultrasonography showed that the enlarged fistula connected to the tumor (Fig. 1b). A 15 × 15-mm tumor was observed at the former drainage site (Fig. 2). A biopsy taken from the tumor revealed a mucinous adenocarcinoma. We diagnosed the tumor as FAMC. Tumor markers, including carcinoembryonic antigen and carbohydrate antigen 19-9 levels, were within the reference range. Colonoscopic evaluation did not reveal other synchronous colorectal cancer. [18F]-fluoro-2-deoxy-d-glucose-positron emission tomography/computed tomography revealed pelvic and inguinal lymph node metastasis (Fig. 1c). No distant organ metastases were observed. Laparoscopic abdominoperineal resection with pelvic and inguinal lymph node dissection was performed. Because paraaortic lymph node metastasis was identified intraoperatively, a curative resection could not be performed. The operative time was 489 min, and the blood loss was 227 mL. She had transient unilateral obturator nerve paralysis and required rehabilitation. She was discharged on postoperative day 23. The tumor was protruding from the surface of the anal canal (Fig. 3) Histopathology of the resected specimen showed poorly differentiated mucinous adenocarcinoma proliferated around the distal aspect of the anal fistula (Fig. 4a, b). The lumen of the anal fistula was covered with highly dysplastic cells (Fig. 4c).

Genetic analyses confirmed the KRAS mutations in the primary tumor. Chemotherapy for paraaortic lymph node metastasis was given with fluorouracil, leucovorin, oxaliplatin, and irinotecan with bevacizumab according to the colorectal adenocarcinoma regimen [6–8]. At 23 months after the surgery, lymph node metastases have shrunk and there is no evidence of relapse.

## Discussion

Chronic inflammation in organs is one of the causes of cancer development and proliferation [1, 2]. More than 10 years after the onset of anal fistula, the risk of developing FAMC increases [3–5]. However, we experienced a case of FAMC which was diagnosed only 3 years after the treatment of a perianal abscess. The findings of transanal ultrasonography and pathological examination suggested that epithelial cell dysplasia and subsequent carcinogenesis might have already progressed prior to the development of the perianal abscess. Consequently, FAMC can arise from anal fistulas in fewer than 10 years.

Three criteria are proposed to diagnose FAMC: (1) a preexistence of the fistula for longer than 10 years, (2) no carcinoma in the proximal side of the fistula, and (3) lack of involvement of the internal opening of the fistula [1–3]. Chronic inflammation caused by a long-standing anal fistula can predispose to carcinogenesis and promote cancer development. However, the basic rationale for setting the duration of the anal fistula at 10 years or longer is unclear.

FAMC can arise from anal fistulas present for fewer than 10 years. The Japanese multi-institutional research reported that, of the 164 patients with anal fistula-associated carcinoma, 60 patients (37%) had a history of anal fistula for fewer than 10 years [9]. A single-institutional review in the US showed that three (21%) of 14 anal fistula-associated carcinomas developed within 10 years after the diagnosis of anal fistula [10].

It is possible that their short disease period is responsible for the delayed diagnosis of their anal fistulas. Some patients might take a long time to present because of unawareness of symptoms. Other patients might hesitate to visit the hospital because of anxiety or shame, even if they were already aware of symptoms. The onset of anal fistula could be recognized later than its actual onset, when determined retrospectively based on medical records. Delayed diagnosis of the anal fistula may account for the short disease period in patients with FAMC.

The short disease period in our patient, however, was not related to delayed diagnosis of anal fistula, because we did not observe any anal fistula at the time of the treatment for the perianal abscess. This means that the FAMC proliferated within 3 years. It is illogical to assume that an anal fistula formed after treatment of a perianal abscess and then carcinogenesis occurred within 3 years. Based on transanal ultrasonographic as well as the pathological findings, we presumed that the development of dysplasia and carcinoma had preceded the development of the perianal abscess.

The transanal ultrasonography showed that a fistula-like structure in the intersphincteric layer had already existed at the time of development of the perianal abscess, indicating that latent chronic inflammation had also been there, though the patient did not have any symptoms of the disease. Dysplasia and carcinogenesis of perianal epithelial cells could proceed in the context of that latent chronic inflammation.

Histopathological examination showed that highly dysplastic cells lined the lumen of the anal fistula, and FAMC had proliferated around the drainage site of the perianal abscess. These findings supported the contention that the dysplastic cells developed from the epithelial cells of the fistula and replaced them. It is possible that FAMC cells arise among these dysplastic cells and floating FAMC cells in the abscess were transplanted to the drainage site. Though the primary FAMC was not found in the fistula histopathologically, it might be, because the primary lesion was too small to be found, or it had already been involved in the transplanted FAMC.

Ninety percent of anal fistulas are considered to arise from cryptoglandular infection [3]. According to cryptoglandular infectious theory, cryptitis followed by perianal abscess and anal fistula are continuous processes in the same infectious disease. Hence, the beginning of the perianal inflammation-associated anal fistulas should be regarded as the onset of cryptitis rather than the development of anal fistulas. The progression of each step is different depending on the case. Proctologists often encounter patients who develop anal fistula after experiencing several episodes of perianal abscess formation over the years. Those patients have had chronic inflammation before the development of anal fistulas, which can lead to malignant transformation of perianal epithelial cells.

Histopathological evidence of carcinoma is essential for preoperative assessment. However, concerning FAMC, documenting carcinomatous cells via biopsy is often difficult because of the relatively small size of the carcinomatous component in the tumor compared with the huge amount of the mucinous component [11]. Even when the first biopsy is negative, a repeat biopsy is recommended to avoid misdiagnosis [12]. In addition, comprehensive assessment including cytology of the mucin components and imaging studies should be considered [11, 13]. Meticulous examinations should be conducted when the anal fistula is suspected of being associated with FAMC. Even if the duration of the anal fistula is fewer than 10 years, the possibility of FAMC should not be excluded. The bias that anal fistula-associated carcinoma always arises from a long-standing anal fistula can risk losing an opportunity for early diagnosis of FAMC.

In addition, the patient dropped out of follow-up after the perianal abscess drainage, which might have led to overlooking surgical wound complications. Unhealed wounds, mucinous secretion, and early induration, which were observed in the surgical site of the perianal abscess and anal fistula, might be related to FAMC [14]. If we had performed a complete follow-up after treatment, we might have noticed wound complications and detected FAMC earlier. We believe that following up patients until complete wound healing after treatment for a perianal abscess is necessary to avoid missing an opportunity to detect latent FAMC.

## Conclusion

We experienced a case of mucinous adenocarcinoma developing in the anal canal 3 years after the treatment of a perianal abscess. Cryptitis and perianal abscesses that precede anal fistula development may promote dysplasia and carcinogenesis of perianal epithelial cells. It should be considered that FAMC can develop even in patients with anal fistula duration fewer than 10 years.

## Data Availability

All data generated or analyzed during this investigation are included in the published manuscript.

## Abbreviations

FAMC: Fistula-associated mucinous adenocarcinoma
FDG: [18F]-fluoro-2-deoxy-d-glucose
H&E: Hematoxylin and eosin
